# First person – Deepika Vasudevan

**DOI:** 10.1242/dmm.049431

**Published:** 2022-03-16

## Abstract

First Person is a series of interviews with the first authors of a selection of papers published in Disease Models & Mechanisms, helping early-career researchers promote themselves alongside their papers. Deepika Vasudevan is first author on ‘ A protein-trap allele reveals roles for *Drosophila* ATF4 in photoreceptor degeneration, oogenesis and wing development’, published in DMM. Deepika conducted the research described in this article while a postdoctoral fellow in Hyung Don Ryoo's lab at New York University Grossman School of Medicine, New York, NY, USA, and is now an assistant professor at University of Pittsburgh School of Medicine, Pittsburgh, PA, USA, investigating cellular stress responses in disease and development.


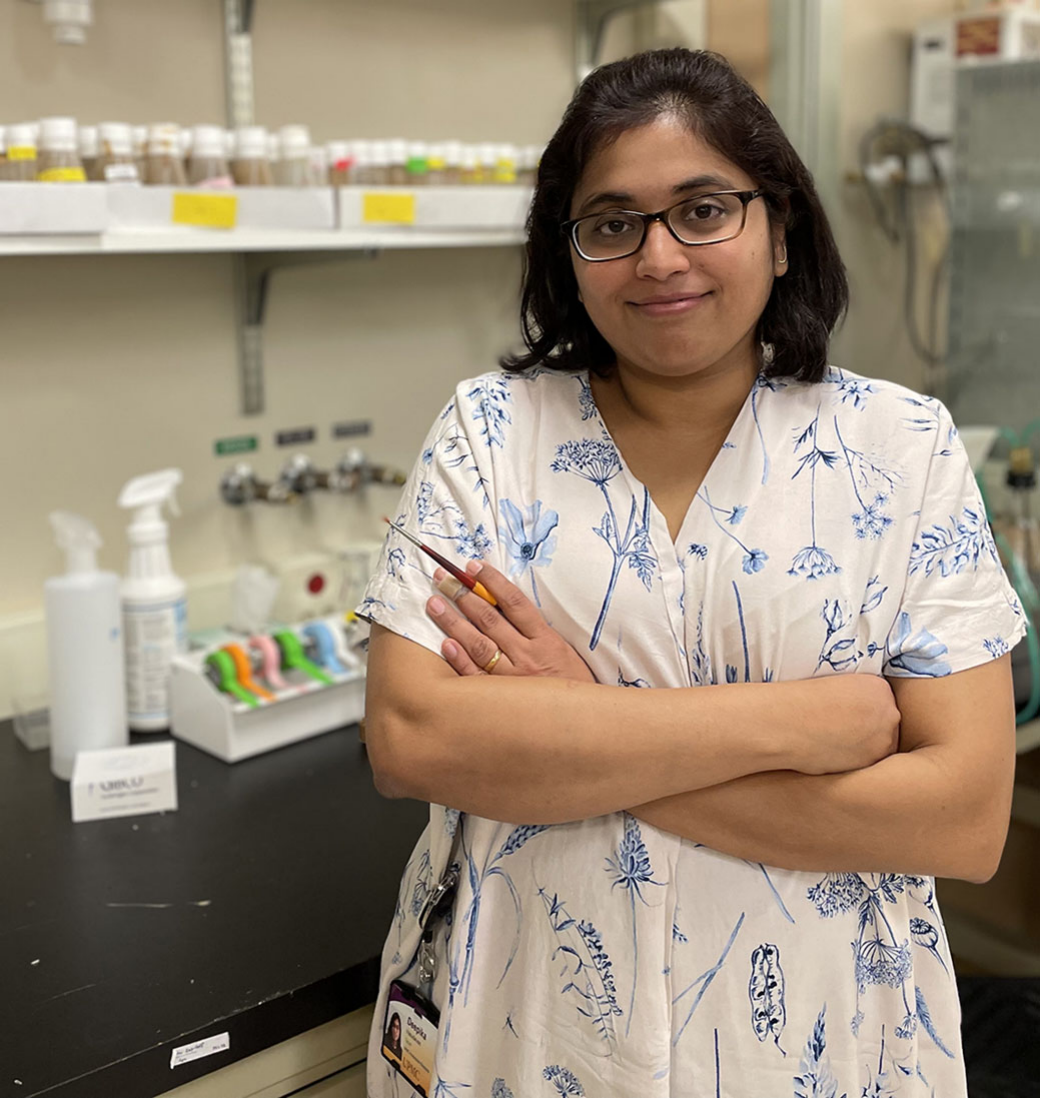


**Deepika Vasudevan**

**How would you explain the main findings of your paper to non-scientific family and friends?**

All organisms have evolved mechanisms to cope with stresses such as nutrient deprivation or infection. Turns out these mechanisms are not only essential to help maintain organismal function during stress conditions, but also during development and normal tissue function. Using fruit flies bearing a mutation in a stress response factor, *crc* (*ATF4* in humans), we see that the stress response mechanisms are required for proper function of neurons in the eye and in maturation of eggs (oogenesis). We also found that loss of *crc* exacerbates degeneration of retinas in a fruit fly model for a human disease called retinitis pigmentosa.

**What are the potential implications of these results for your field of research?**

The role of *crc* in development has largely been limited to larval/pupal stages because that's when the previously available mutants survive until. We were lucky to find a mutant that makes it to adulthood, which has revealed all new roles for *crc* that we didn't know about before! Our paper is a report of sorts for the phenotypes we saw, but it opens up new avenues of study for both researchers in the stress response field and those interested in vision/oogenesis.

“The genetic tools available in fruit flies (*Drosophila*) are unbeatable and our work is exemplary of that.”

**What are the main advantages and drawbacks of the model system you have used as it relates to the disease you are investigating?**

The genetic tools available in fruit flies (*Drosophila*) are unbeatable and our work is exemplary of that. The *crc* allele we use in the paper acts as a mutant when homozygous but as a reporter for *crc* gene expression when heterozygous. The best part is that we didn't even make this allele! The fly community is an exceptionally collaborative and cooperative venture, and researchers in Baylor College of Medicine actually made this mutant (as part of a larger library) and shared it with the community.

As much as I love the fly model, there are limitations to how much human physiology we can model in this system. So while the fly is a great discovery platform, observations made in flies often require validation and further examination in vertebrates for pharmacological application such as therapeutic interventions for retinitis pigmentosa.

**Figure:**
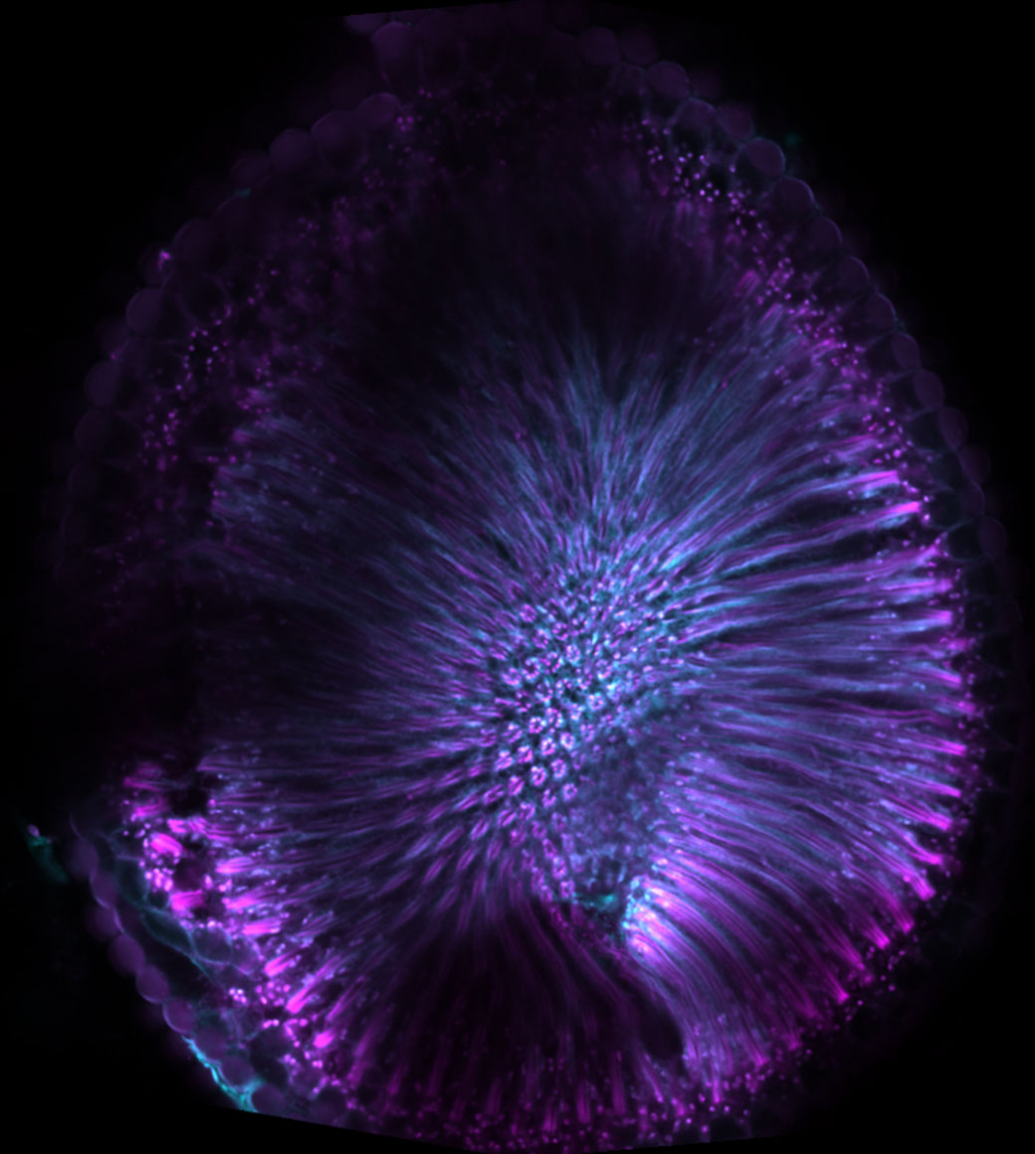
Confocal image of a 20-day-old *Drosophila* eye showing individual ommatidial structures that have started to degenerate (Tubulin in magenta, Rhodopsin 1 in cyan).

**What has surprised you the most while conducting your research?**

Of all the work as I did as a postdoc, the work in this paper was my most intellectually independent venture. I worked in collaboration with another postdoc (Hidetaka) and an undergraduate trainee (Grace) in the lab, both of whom are authors on this paper. In our regular meetings we were all struck by how consistent the phenotypes we found were: Hidetaka, who characterized the wing phenotypes also noticed a striking fertility defect; Grace, who was looking at eye phenotypes consistently saw wing defects, and so on. This might not seem noteworthy to many, but it was remarkable to us given how often we come across phenotypes that are not fully penetrant. So in addition to boosting our confidence in the data, the robustness of our phenotypes made for an extremely cooperative and fun project.

**Describe what you think is the most significant challenge impacting your research at this time and how will this be addressed over the next 10 years?**

Model organism research can be a powerful discovery platform for human biomedical applications; however, it also simply expands the frontiers of scientific knowledge. As someone who has been funded by the National Institutes of Health for a substantial portion of my career so far (and hopefully for the foreseeable future!), I find myself performing a balancing act between curiosity-driven versus human biology-driven research using *Drosophila*. To me these are both equally valuable pursuits, but my sense is that there is polarization in the scientific community as to what type of research should be prioritized, and this is ultimately reflected in what type of research is funded. For example, it continues to surprise me how few vertebrate immunologists know that innate immune pathway components were first discovered in *Drosophila*! While broader science communication efforts are remedying this, I hope academic institutions take a more active role in showcasing the complementary nature of basic and translational research.

“[…] mentoring needs to be incentivized more than it is currently.”

**What changes do you think could improve the professional lives of early-career scientists?**

I think a huge part of the proverbial ‘leaky pipeline’ problem is the lack of accountability for mentors/advisors. I have been very fortunate to have extremely supportive PhD and postdoc advisors, but I know from first- and second-hand experiences that they are not the norm. Given the sheer amount of influence that advisors have on a trainee's career, I think mentoring needs to be incentivized more than it is currently. My favourite idea is that funding agencies ought to require ‘reference letters’ from trainees as part of grant applications which utilize trainee efforts – this can be implemented at every level, i.e. undergraduate, graduate, postdoc and new PI. I believe this will substantially improve accountability and facilitate better trainee-mentor communication.

**What's next for you?**

We submitted this work as I was finishing up my postdoc training in Don Ryoo's lab at New York University Grossman School of Medicine. I started as an assistant professor in the Department of Cell Biology at the University of Pittsburgh School of Medicine 2 weeks after submission! It's been an incredibly fun experience so far setting up a lab, building a team and exploring new research avenues (check out www.flystresslab.com). One of our most exciting projects is to try and figure out the mechanism of the female fertility phenotypes described in this work. With a bit of luck, we are hoping to report our findings in a year or so!
